# The Paired Siglecs in Brain Tumours Therapy: The Immunomodulatory Effect of Dexamethasone and Temozolomide in Human Glioma In Vitro Model

**DOI:** 10.3390/ijms22041791

**Published:** 2021-02-11

**Authors:** Przemyslaw Wielgat, Natalia Wawrusiewicz-Kurylonek, Robert Czarnomysy, Karol Rogowski, Krzysztof Bielawski, Halina Car

**Affiliations:** 1 Department of Clinical Pharmacology, Medical University of Bialystok, Waszyngtona 15A, 15-274 Bialystok, Poland; hcar@umb.edu.pl; 2 Department of Clinical Genetics, Medical University of Bialystok, Waszyngtona 13, 15-089 Bialystok, Poland; natalia.wawrusiewicz-kurylonek@umb.edu.pl; 3 Department of Synthesis and Technology of Drugs, Medical University of Bialystok, Kilińskiego 1, 15-089 Bialystok, Poland; robert.czarnomysy@umb.edu.pl (R.C.); kbiel@umb.edu.pl (K.B.); 4 Department of Experimental Pharmacology, Medical University of Bialystok, Szpitalna 37, 15-295 Bialystok, Poland; karolrogovsky@gmail.com

**Keywords:** Siglec, glioma, microglia, dexamethasone, temozolomide

## Abstract

The paired sialic acid-binding immunoglobulin like lectins (Siglecs) are characterized by similar cellular distribution and ligand recognition but opposing signalling functions attributed to different intracellular sequences. Since sialic acid—Siglec axis are known to control immune homeostasis, the imbalance between activatory and inhibitory mechanisms of glycan-dependent immune control is considered to promote pathology. The role of sialylation in cancer is described, however, its importance in immune regulation in gliomas is not fully understood. The experimental and clinical observation suggest that dexamethasone (Dex) and temozolomide (TMZ), used in the glioma management, alter the immunity within the tumour microenvironment. Using glioma-microglia/monocytes transwell co-cultures, we investigated modulatory action of Dex/TMZ on paired Siglecs. Based on real-time PCR and flow cytometry, we found changes in SIGLEC genes and their products. These effects were accompanied by altered cytokine profile and immune cells phenotype switching measured by arginases expression. Additionally, the exposure to Dex or TMZ increased the binding of inhibitory Siglec-5 and Siglec-11 fusion proteins to glioma cells. Our study suggests that the therapy-induced modulation of the interplay between sialoglycans and paired Siglecs, dependently on patient’s phenotype, is of particular signification in the immune surveillance in the glioma management and may be useful in glioma patient’s therapy plan verification.

## 1. Introduction

Gliomas are a unique class of human intracranial malignancies with multiple therapeutic difficulties due to their biological diversity and intense interplay with the structural and functional components of the microenvironment [1,2]. The tumour-controlled extracellular communication is critical to promote the progression switch mechanisms and diminish antitumor processes, including immune surveillance [3,4]. In response to the tumour-derived factors, both, resident and peripheral immune cells, including CD4^+^ regulatory T cells (Tregs), eosinophiles, monocytes and resident microglia, undergo reprogramming that results in altered secretory capacity and phagocytic functions [4,5,6,7]. The crosstalk between glioma cells and the heterogenic immune population is mediated by suppressive cytokines that disturb the balance of proliferation and apoptosis, and switch the activity phenotype from M1 to M2. Accumulating data suggest that tumours employ regulatory cell membrane protein systems to evade immune cell attacks [8]. Immune checkpoints, the major guardians of immune homeostasis, play a critical role in self-tolerance processes underlying the autoimmunity prevention mechanisms. However, the cancer-related molecular mimicry of the host checkpoint systems interferes with the self-tissue antigens and screw the activation status towards immunosuppression and tolerance [9,10,11].

The cell membrane sialic acids play critical role as a regulators of adhesion-related cell-cell interaction underlying immune recognition [12,13]. The aberrantly sialylated tumour-specific glycotopes reduce cancer immunogenicity by the hiding of cell surface antigens, however, the tumour immune evasion is mainly facilitated by immune receptor families, such as the most of CD33-related sialic acid-binding immunoglobulin like lectins (Siglecs), that recognize cancer sialoglycans and transmit suppressive signals [14,15]. In the brain, the binding of polysialic acid (PSA) with microglial Siglec-11 receptor seem to be closely associated with the restriction of the immune function initiated by immunoreceptor tyrosine-based inhibitory motif (ITIM)-coupled signalling pathway [16,17]. Among the Siglec family members, the paired receptors, Siglec-5/14 and Siglec-11/16, display extremely similar extracellular ligand-binding regions and comparable cellular distribution resulting in the same ligand recognition ability whereas the diverse intracellular signalling pathways trigger the opposite effects. While the expression of inhibitory receptors was described in several human pathologies, the role of activatory counterparts is not fully understood [18]. However, recent advances in glycoimmunology suggest that targeting Siglec-sialoglycans checkpoint axis might be useful in the control of cancer immune evasion [19,20].

Despite the progress in molecular immunotherapy, conventional drugs are the strategy of choice for primary and recurrent gliomas in the routine clinical practice. Temozolomide (TMZ) and dexamethasone (Dex) are commonly administered in the management of high-grade gliomas. Temozolomide (TMZ) and dexamethasone (Dex) are commonly administered in the management of high-grade gliomas. According to the National Comprehensive Cancer Network guidelines (NCCN Guidelines) and the recommendations of the European Society for Medical Oncology (ESMO), TMZ is the most widely used and effective alkylating chemotherapeutic agent resulting in the high cytotoxicity and apoptosis within the glial tumour mass [21,22]. These effects are enhanced during concomitant radiotherapy and TMZ administration as shown in studies by the European Organization for the Research and Treatment of Cancer and the National Cancer Institute of Canada Clinical Trials Group (EORTC/NCIC) [23,24]. Besides the apoptotic effects in glioma cells, the dosing of temozolomide may impact Tregs numbers and function and thereby enhance the efficacy of further immunotherapy. Surprisingly, TMZ concomitantly with steroids and/or radiotherapy induces strong immunosuppression by lowering the absolute lymphocyte count, however, the effects on monocyte/macrophages and microglia subsets remain not fully understood [25]. In particular, Dex-based glioma management includes multiple pitfalls in the central nervous system (CNS) associated with strong effects on immune surveillance in the brain, however, the molecular mechanisms of this phenomenon are not fully understood [23,24]. The previous studies revealed that dexamethasone, the widely used anti-oedemic agent in patients with glioma, influences on Siglec-sialic acid axis and thereby may induce the immune inversion in microglia [26,27]. Based on these observations, we developed in vitro co-culture model to evaluate the role of interplay between Siglec receptors and cell membrane sialoglycans in the immune regulation in response to conventional glioma therapy. The glioma cells of different sensitivity to TMZ and monocytic THP-1 or microglial HMC3 cells were cultivated in the transwell co-culture systems or monocultures and exposed to TMZ/Dex treatment. Since the immune evasion is known as cancer progression promoting mechanism, the immune phenotyping of patients by the engagement of several immune checkpoint axis can be of clinical importance in the context of efficiency standard cancer therapy.

## 2. Results

### 2.1. The Immune Status of THP-1 and HMC3 Cells in Mono- and Co-Cultures Exposed to Dex/TMZ Treatment

**Figure 1:**
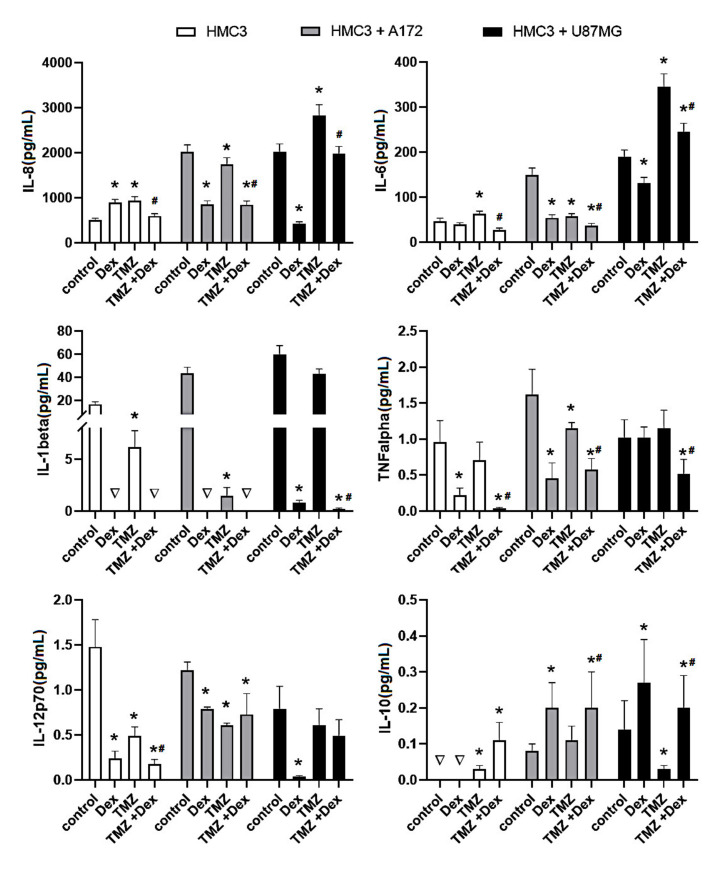
Flow cytometric analysis of population of HMC3 cells for expression of IL-8, IL-6, IL-1β, TNFα, IL-12p70 and IL-10. Cells were grown in monoculture or co-culture and exposed to Dex (10 μM) and/or TMZ (10 μM) for 24 h. The immune cell samples were collected and stained with phycoerythrin-conjugated antibodies for expression of selected cytokines. The concentration of the target proteins was determined using the standard curve according to the manufacturer instructions. * *p* < 0.05 vs. corresponding control group, # *p* < 0.05 TMZ + Dex vs. TMZ treated group; ∇—concentration <0.01 pg/mL. Control—nonstimulated cells, Dex—dexamethasone alone treated cells, TMZ—temozolomide alone treated cells, TMZ + Dex—cells treated with temozolomide in combination with dexamethasone.

**Figure 2:**
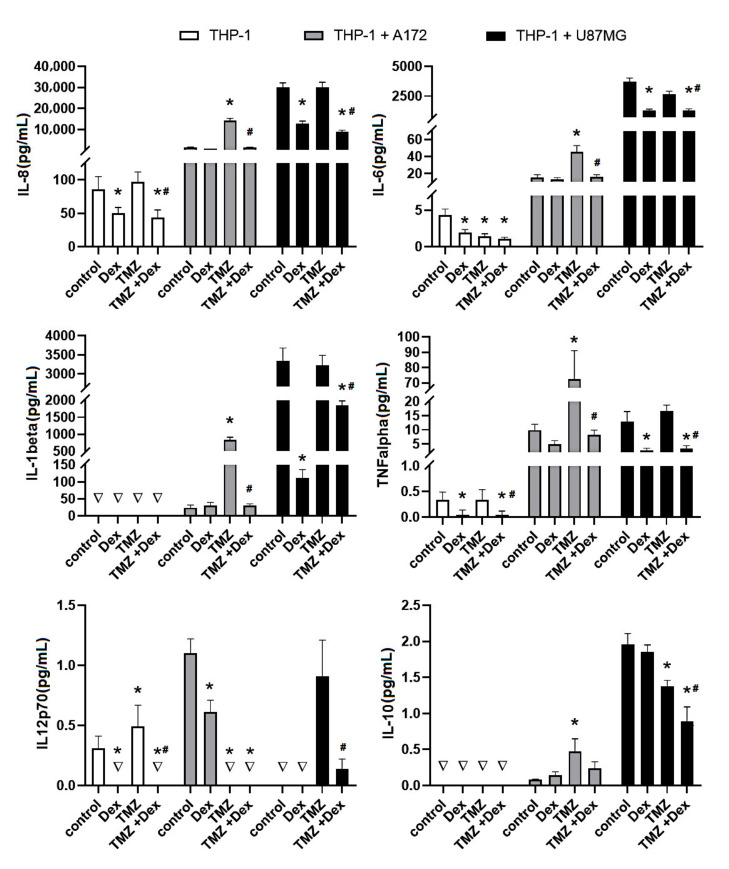
Flow cytometric analysis of population of THP-1 cells for expression of IL-8, IL-6, IL-1β, TNFα, IL-12p70 and IL-10. Cells were grown in monoculture or co-culture and exposed to Dex (10 μM) and/or TMZ (10 μM) for 24 h. The immune cell samples were collected and stained with phycoerythrin-conjugated antibodies for expression of selected cytokines. The concentration of the target proteins was determined using the standard curve according to the manufacturer instructions. * *p* < 0.05 vs. corresponding control group; # *p* < 0.05 TMZ + Dex vs. TMZ treated group; ∇—concentration <0.01 pg/mL. Control—nonstimulated cells, Dex—dexamethasone alone treated cells, TMZ—temozolomide alone treated cells, TMZ + Dex—cells treated with temozolomide in combination with dexamethasone.

To assess the immune status of human THP-1 and HMC3 cells in response to Dex and/or TMZ, levels of representative cytokines were studied by flow cytometry. The analysis of IL-8, IL-6, IL-1β, IL-12p70, IL-10 and TNFα protein levels, expressed as a concentration in cellular supernatants evidenced differences between different cell culture setups (THP-1 and HMC3 monocultures and THP-1/A172, HMC3/A172, THP-1/U87MG and HMC3/U87MG co-cultures). Firstly, the levels of selected cytokines in the co-cultured cells were higher than those tested in monoculture setups. However, in the case of THP-1 cells in monoculture, the concentration of IL-1β, IL-10 and IL-12p70 was below 0.01 pg/mL. No rational explanation is available for this observation and additional studies are required to explain this effect. Secondly, the immune response of the THP-1-based co-cultures was extremely higher when compared to the HMC3/A172 and HMC3/U87MG co-culture setups. The analysis of cytokines levels upon exposure to Dex and/or TMZ evidenced differences between groups, but precisely quantifiable results were received only for IL-8, IL-6, IL-10 and TNFα. As expected, the inhibitory effect of Dex on the concentration of IL-6, IL-8 and TNFα has been observed in THP-1 mono- and co-cultures. In contrast, HMC3 monocultures, but not co-cultures, showed significantly enhanced level of IL-8 (892 ± 72.3 vs. 503 ± 45.5 naïve control). Additionally, the exposure to TMZ caused changes in immune activity of THP-1 and HMC3 cells. In response to TMZ, the level of IL-8, IL-6 and TNFα was significantly higher in THP-1/A172 co-cultures, whereas the opposite effects were observed in HMC3/A172 co-cultures (*p* < 0.05). Interestingly, the expression of IL-8 and IL-6 was extremely increased in HMC3/U87MG co-cultures, whereas alterations in THP-1 monocytes grown in the presence of A172 cells were statistically insignificant when compared to naïve control cells. The TMZ-induced changes in cytokine expression in all analysed culture systems were significantly reduced when Dex concomitantly was used. In the presence of glioma cells, the expression of IL-10 tended to be increased in both THP-1 and HMC3 cells. In HMC3 cell line this effect was significantly strengthened in response to Dex. In both HMC3/A172 and THP-1/U87MG co-cultures, the level of IL-10 was significantly reduced in response to 10 μM TMZ alone or in combination with Dex (Figure 1 and Figure 2).

**Figure 3:**
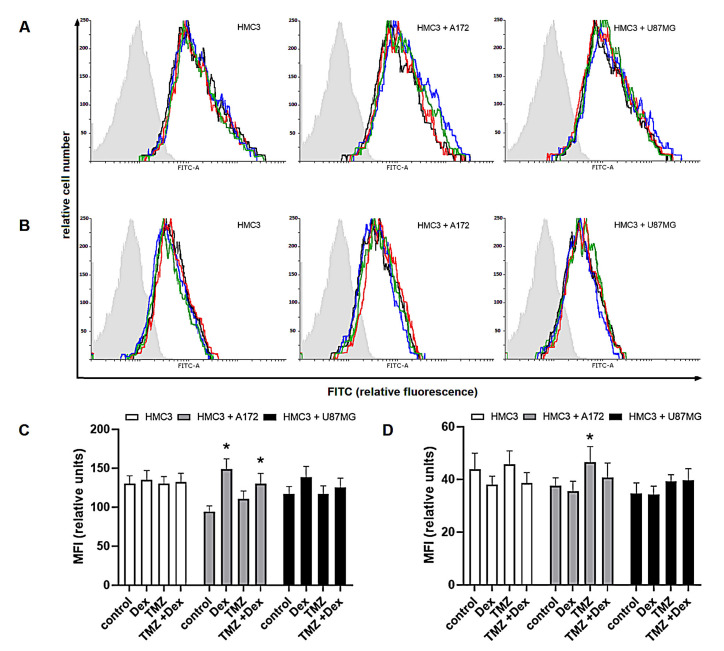
Expression of (**A**,**C**) Arg-1 and (**B**,**D**) Arg-2 in HMC3 cells grown in monoculture and co-culture and exposed to Dex and/or TMZ. Representative histograms were derived from flow cytometric analysis of 5000 cells and show isotype control (light grey histogram), control cells (black line) Dex-treated cells (blue line), TMZ-treated cells (red line) and cells concomitantly treated with Dex/TMZ (green line). Data are presented as a median fluorescence intensity (MFI) from at least three independent experiments done in duplicate; * *p* < 0.05 vs. corresponding control group.

**Figure 4:**
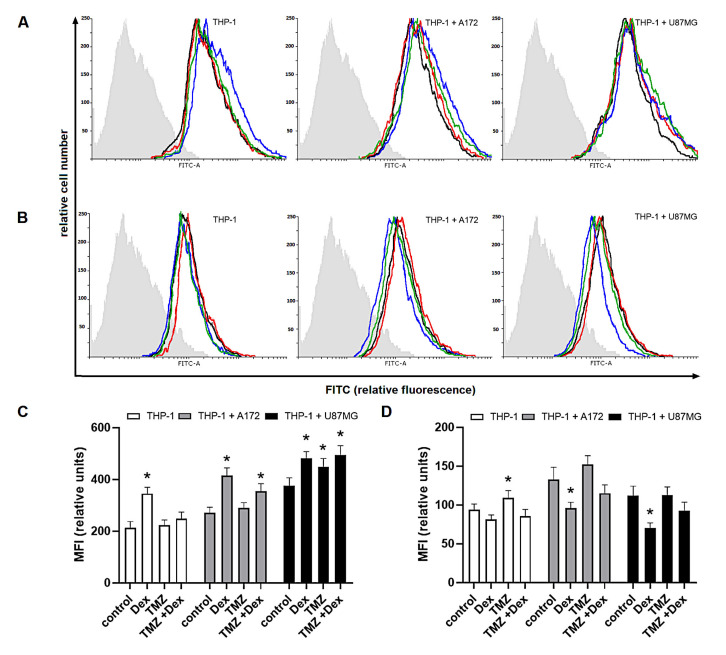
Expression of (**A**,**C**) Arg-1 and (**B**,**D**) Arg-2 in THP-1 cells grown in monoculture and co-culture and exposed to Dex and/or TMZ. Representative histograms were derived from flow cytometric analysis of 10,000 cells and show isotype control (light grey histogram), control cells (black line) Dex-treated cells (blue line), TMZ-treated cells (red line) and cells concomitantly treated with Dex/TMZ (green line). Data are presented as a median fluorescence intensity (MFI) from at least three independent experiments done in duplicate; * *p* < 0.05 vs. corresponding control group.

In the further evaluation of modulatory properties of Dex and/or TMZ, we investigated the intracellular expression of Arg-1 and Arg-2 known as markers of M2 and M1 immune phenotype, respectively. In THP-1 monoculture, the expression of Arg-1 was significantly increased in response to Dex (MFI: 345 ± 24.8 vs. 212 ± 25.3 naïve cells), whereas exposure to 10 μM TMZ resulted in significant increase of Arg-2 expression (MFI: 109 ± 9.4 vs. 93.9 ± 7.2 naïve control). In HMC3 monocultures, changes in both Arg-1 and Arg-2 expression were statistically insignificant in all experimental groups. In both, THP-1/A172 and HMC3/A172 co-cultures, the expression of Arg-1 was significantly increased in response to Dex alone (MFI: 414 ± 30.8 vs. 271 ± 21.8 naïve co-culture) or in combination with TMZ (355 ± 28.8 vs. 271 ± 21.3 naïve co-culture). Hence, the exposure to TMZ in these systems was associated with altered expression of Arg-2 (MFI; THP-1/A172: 152 ± 11.4 vs. 133 ± 15.5 naïve co-culture, *p* > 0.05; HMC3/A172: 46.56 ± 5.9 vs. 37.5 ± 3.05 naïve co-culture, *p* < 0.05). In THP-1/U87MG co-culture, significant increase in Arg-1 expression was observed in response to Dex (MFI: 482 ± 25.8 vs. 375 ± 31.3 naïve co-culture) and TMZ alone (MFI: 449 ± 32.4 vs. 375 ± 31.3 naïve co-culture) or in concomitant treatment (MFI: 495 ± 35.5 vs. 375 ± 31.3 naïve co-culture). Furthermore, the expression of Arg-2 was significantly reduced in response to Dex when compared to unstimulated co-culture (MFI: 70.41 ± 6.5 vs. 112 ± 12.2). Surprisingly, there were not significant alterations in Arg-1 and Arg-2 expression in HMC3/U87 co-cultures (Figure 3 and Figure 4).

### 2.2. The Dex/TMZ—Related Changes in Siglecs Expression in Human THP-1 and HMC3 Cells

**Figure 5:**
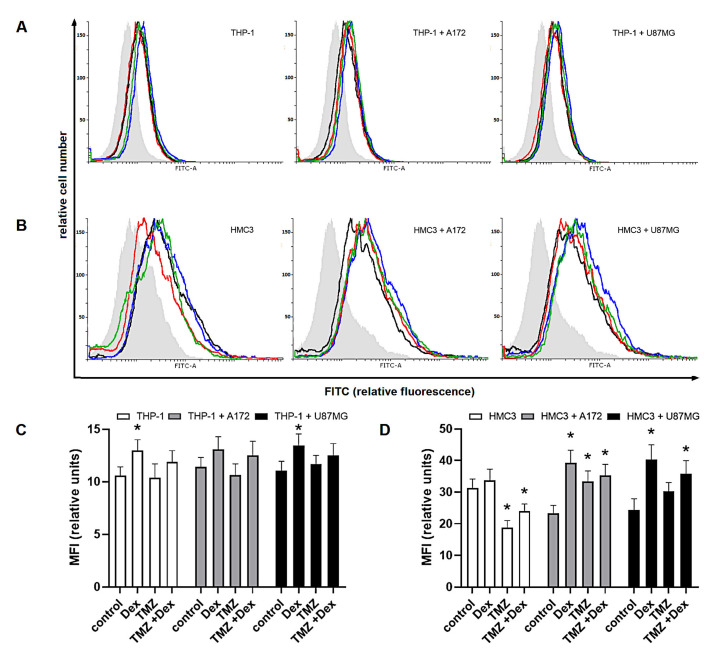
Expression of (**A**,**C**) Siglec-5 in human THP-1 cells and (**B**,**D**) Siglec-11 in human HMC3 cells grown in monoculture and co-culture exposed to Dex and/or TMZ. Representative histograms were derived from flow cytometric analysis of 13,000 cells and show isotype control (light grey histogram), control cells (black line) Dex-treated cells (blue line), TMZ-treated cells (red line) and cells concomitantly treated with Dex/TMZ (green line). Data are presented as a median fluorescence intensity (MFI) from at least three independent experiments done in duplicate; * *p* < 0.05 vs. corresponding control group.

As shown, the flow cytometric analysis with specific antibodies confirmed the expression of Siglec-5 and Siglec-11 in THP-1 and HMC3 cells, respectively. The expression of Siglec-5 protein in monoculture THP-1 control cells was similar to those detected in both naïve co-culture systems (HMC3/A172: 11.4 ± 0.9 vs. 10.57 ± 0.85 HMC3; HMC3/U87: 11.04 ± 0.9 vs. 10.57 ± 0.85 HMC3, *p* < 0.05). The amount of Siglec-5 in THP-1 monocultures was significantly elevated in response to 10 μM Dex (Dex: 12.98 ± 1.02 vs. 10.57 ± 0.85 naïve control). The similar tendency was observed when THP-1 cells were kept in the presence of U87MG, but not A172 cells. However, there were no significant changes in response to TMZ alone or in combination with Dex (Figure 5).

The HMC3 cells showed strong reactivity with Siglec-11 antibody. Compared with monoculture control, the amount of Siglec-11 protein was lower in both naïve co-culture systems (HMC3/A172: 23.29 ± 2.54 vs. 31.34 ± 2.8 HMC3, *p* < 0.05; HMC3/U87: 24.36 ± 3.55 vs. 31.34 ± 2.8 HMC3, *p* < 0.05). In monocultures and co-cultures, the expression of Siglec-11 in HMC3 cells was increased after exposure to Dex (HMC3: 33.68 ± 3.6 vs. 31.34 ± 2.8 naïve control, *p* > 0.05; HMC3/A172: 39.24 ± 4.05 vs. 23.29 ± 2.54 naïve control, *p* < 0.05; HMC3/U87MG: 40.32 ± 4.65 vs. 24.36 ± 3.55 naïve control, *p* < 0.05). We found the opposite effects of TMZ on Siglec-11 in mono- and co-cultured cells. Surprisingly, the expression of Siglec-11 in HMC3 monocultures was significantly reduced in response to TMZ (18.77 ± 2.21 vs. 31.34 ± 2.8 naïve control), but higher when cells were cultivated in transwell system (HMC3/A172: 33.38 ± 3.3 vs. 23.29 ± 2.54 naïve co-culture, *p* < 0.05; HMC-3/U87MG: 30.23 ± 2.8 vs. 24.26 ± 3.55 naïve co-culture, *p* < 0.05). This effect was slightly reversed when TMZ in combination with Dex was used (HMC3: 23.93 ± 2.3 vs. 18.77 ± 2.21 TMZ-treated cells; HMC3/A172: 35.25 ± 3.54 vs. 33.38 ± 3.3 TMZ-treated cells; HMC3/U87MG: 35.87 ± 4.05 vs. 30.23 ± 2.8 TMZ-treated cells; all *p* > 0.05).

### 2.3. Evaluation of Paired Siglecs Genes Expression in Monocytic THP-1 and Micrglial HMC3 Cells Exposed to Dex/TMZ Treatment

**Figure 6:**
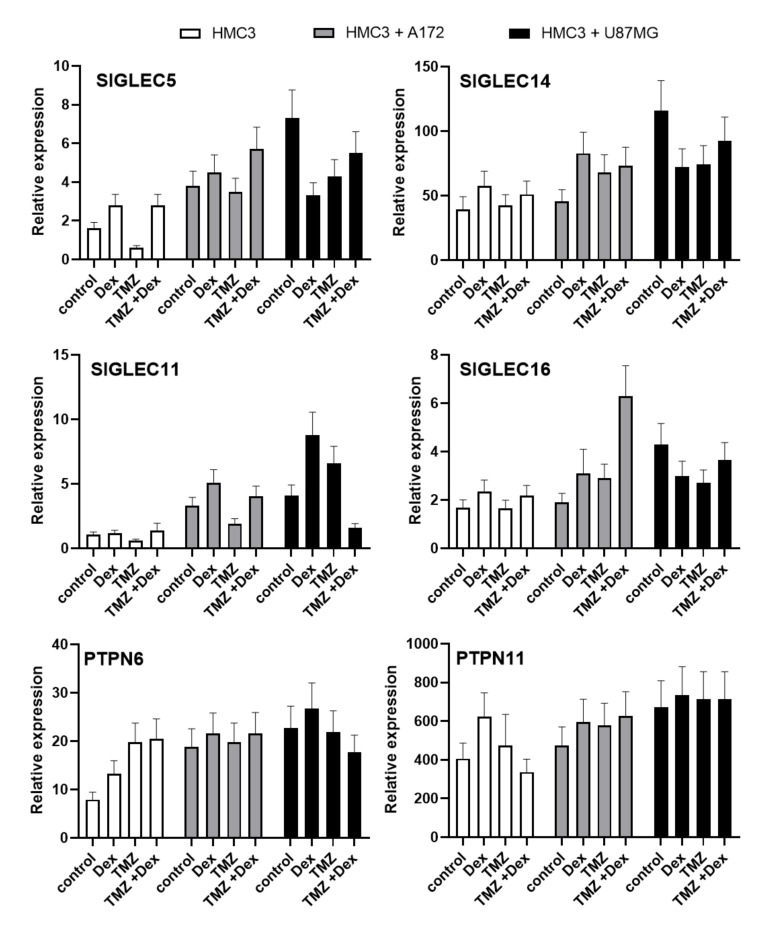
Expression of *SIGLEC5, SIGLEC14, SIGLEC11, SIGLEC16, PTPN6* and *PTPN11* mRNA in HMC3 cells. The analysed transcripts were detected by real time-PCR in HMC3 microglia grown in monoculture and co-culture. The house-keeping gene *GAPDH* was used as an internal loading control. Representative data show a mean of three samples.

**Figure 7:**
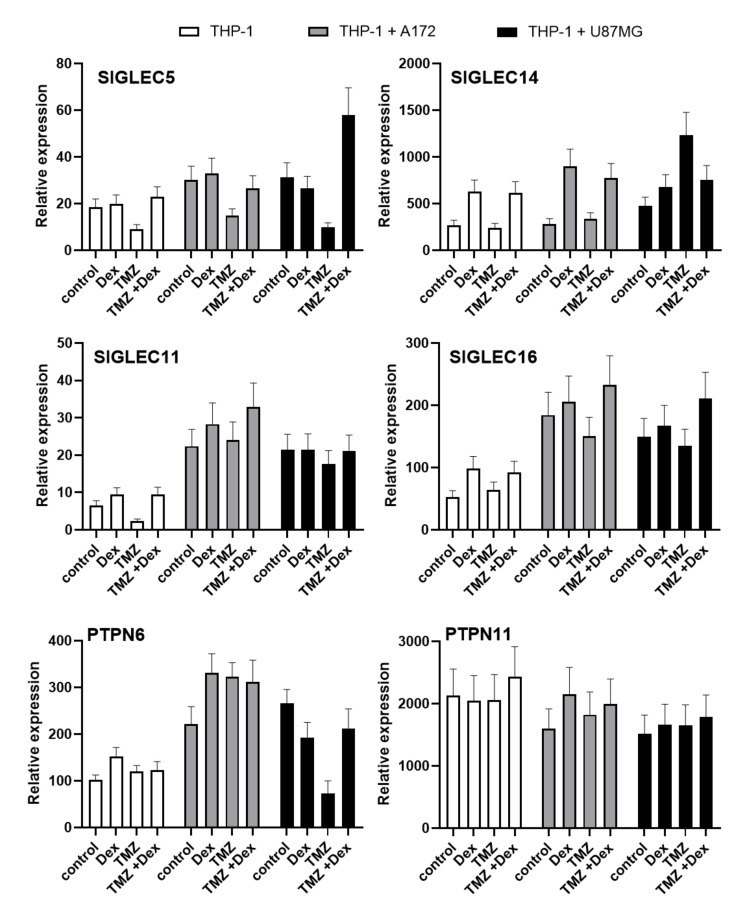
Expression of *SIGLEC5, SIGLEC14, SIGLEC11, SIGLEC16, PTPN6* and *PTPN11* mRNA in THP-1 cells. The analysed transcripts were detected by real time-PCR in THP-1 monocytes grown in monoculture and co-culture. The house-keeping gene *GAPDH* was used as an internal loading control. Representative data show a mean of three samples.

Because the exposure to Dex or TMZ showed the modulatory effect on Siglecs proteins, we performed the preliminary evaluation of transcriptional response induced by these agents. All analysed mRNA transcripts for *SIGLEC* showed higher expression in co-culture systems compared do monocultures. The THP-1 and HMC3 cells stimulated with Dex showed an increased expression of *SIGLEC5*, *SIGLEC*11, *SIGLEC*14 and *SIGLEC*16 mRNA compared to naive cells. The opposite effects were observed when cells were exposed to TMZ. The mRNA transcripts level of *SIGLEC5* and *SIGLEC11* showed a similar tendency in THP-1 and HMC3 cells cultivated in the presence of A172 glioma cells. The mRNA transcripts for *SIGLEC14* and *SIGLEC16* were decreased in THP-1 monocytes exposed to TMZ, but HMC3 microglial cells showed elevated expressions compared to unstimulated control cells (Figure 6 and Figure 7).

In HMC3/U87MG culture system, the levels of mRNA transcripts for *SIGLEC5*, *SIGLEC14* and *SIGLEC16* were decreased in response to Dex or TMZ, whereas *SIGLEC11* was increased. In THP-1 cells grown in the presence of U87MG glioma, the exposure to Dex or TMZ caused decreased *SIGLEC5* transcripts levels, but increased expression of SIGLEC14. The expression of *SIGLEC16* transcripts was increased in response to Dex and decreased after treatment with TMZ. Moreover, *SIGLEC11* transcripts demonstrated similar levels in naive and stimulated cells. Interestingly, Dex counteracted effects of TMZ on *SIGLECs* transcripts expression in combined treatment in all analysed groups. To evaluate the importance of changes in *SIGLECs* transcripts, we performed the expression analysis of genes encoding signalling molecules functionally linked to the Siglec-mediated pathways. The expression of *TYROBP* transcripts (protein tyrosine binding protein), related to activatory Siglecs-mediated signalling pathways, was extremely low in both THP-1 and HMC3 cells (data not shown). The expression of *PTPN6* transcripts (tyrosine-protein phosphatase non receptor type 6, *SHP1*) in HMC3 and THP-1 monocultures tended to be increased in response to Dex or TMZ exposure. Similar effects have been observed when immune cells were cultured with A172 cells. However, THP-1 and HMC3 cells cultured in presence U87MG glioma demonstrated the opposite action of Dex and TMZ on *PTPN6* transcript expression. Interestingly, the THP-1 and HMC3 co-cultures showed a high level of *PTPN11* transcripts (tyrosine-protein phosphatase non receptor type 11, *SHP2*), however, we did not find marked differences between the groups treated with Dex and/or TMZ.

### 2.4. Human Glioma Cells Are Recognized by a Siglec-5/Fc and Siglec-11/Fc Fusion Proteins in Response to Dex/TMZ Treatment

**Figure 8:**
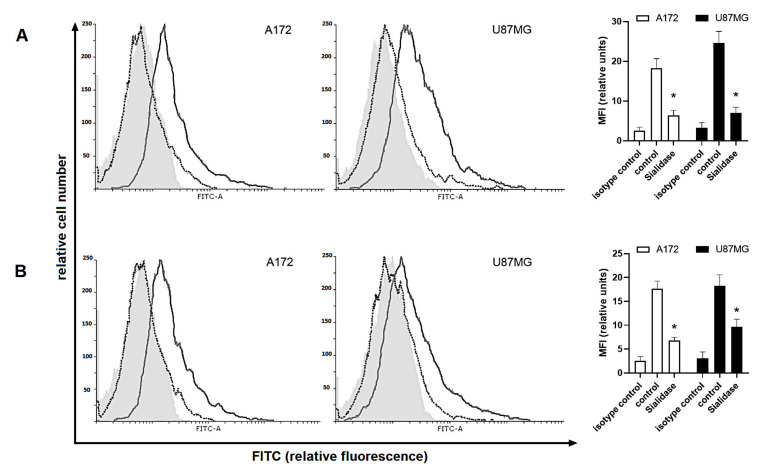
Binding of (**A**) Siglec-5/Fc and (**B**) Siglec-11/Fc fusion proteins to glioma cells digested with α2-3,6,8-neuraminidase. Representative histograms and corresponding bar graphs show isotype control (light grey filled histogram), control cells (grey line) and sialidase—treated cells (black dotted line). Data are presented as a median fluorescence intensity (MFI) from at least three independent experiments done in duplicate; * *p* < 0.05 vs. corresponding control group.

To establish the sialylation-related changes in cell surface glycocalyx and their importance in immune regulation, glioma cells were incubated with the Siglecs fusion proteins featured with high binding preference of specific sialic acids. The fusion proteins of Siglec-5 and Siglec-11 are constructive products of extracellular domains of Siglec-5 and Siglec-11 linking to the FC region of immunoglobulin G (IgG). Both monocultures of A172 and U87MG glioma cells were able to interact with fusion proteins, however, this effect was significantly diminished in cells digested with sialidase (Figure 8).

**Figure 9:**
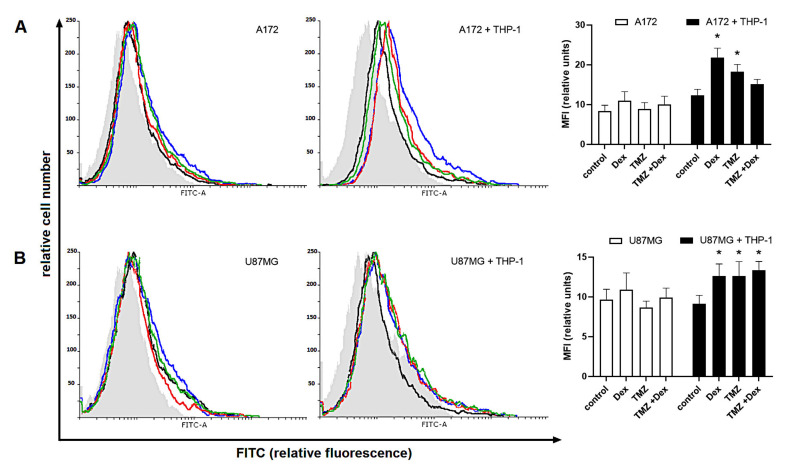
Effect of Dex and/or TMZ on Siglec-5/Fc fusion protein binding to (**A**) A172 and (**B**) U87MG glioma cells grown in monocultures or co-cultures. Representative histograms and corresponding bar graphs show isotype control (light grey filled histogram), control cells (black line), Dex-treated cells (blue line), TMZ-treated cells (red line) and cells concomitantly treated with Dex/TMZ (green line). Data are presented as a median fluorescence intensity (MFI) from at least three independent experiments done in duplicate; * *p* < 0.05 vs. corresponding control group.

**Figure 10:**
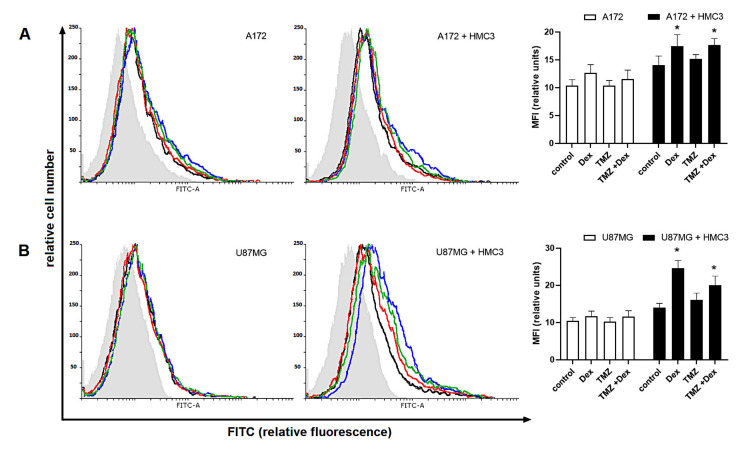
Effect of Dex and/or TMZ on Siglec-11/Fc fusion protein binding to (**A**) A172 and (**B**) U87MG glioma cells grown in monocultures or co-cultures. Representative histograms and corresponding bar graphs show isotype control (light grey filled histogram), control cells (black line), Dex-treated cells (blue line), TMZ-treated cells (red line) and cells concomitantly treated with Dex/TMZ (green line). Data are presented as a median fluorescence intensity (MFI) from at least three independent experiments done in duplicate; * *p* < 0.05 vs. corresponding control group.

Interestingly, the binding of extracellular Siglec domains was insignificantly modulated in response to Dex and/or TMZ. When A172, but not U87MG cells, were co-cultured with THP-1 the affinity of Siglec-5/Fc fusion protein tended to be increased when compared to naive monocultures (A172/THP-1: 12.3 ± 1.5 vs. 8.3 ± 1.5 monoculture; U87MG/THP-1: 9.14 ± 1.05 vs. 9.65 ± 1.3 monoculture). In co-cultures with HMC3, both A172 and U87MG cells, showed enhanced Siglec-11/Fc binding capacity when compared to monocultured glioma cells (A172/HMC3: 14.07 ± 1.6 vs. 10.3 ± 1.04 monoculture; U87MG/HMC3: 13.95 ± 1.2 vs. 10.46 ± 0.9 monoculture). These effects were strengthened in response to Dex alone or in combination with TMZ. In details, the binding of Siglec-11/Fc fusion protein to cell membrane sialic acid was significantly increased in both A172 cells (Dex: 21.8 ± 2.4 vs. 10.9 ± 2.3 naïve co-culture; Dex/TMZ: 15.1 ± 1.2 vs. 10 ± 2.1 naïve co-culture) and U87MG cells (Dex: 24.6 ± 2.1 vs. 11.65 ± 1.4 naïve co-culture; Dex/TMZ: 16.1 ± 1.8 vs. 10.27 ± 1.1 naïve co-culture) whereas the affinity of Siglec-5/Fc protein was significantly enhanced in U87MG/THP-1 co-cultures (Dex: 12.63 ± 1.5 vs. 10.9 ± 13.34 ± 1.1 vs. 9.9 ± 1.2 naïve co-culture). Surprisingly, the exposure to TMZ alone promoted the binding of Siglec-5/Fc fusion protein in both co-culture systems (*p* < 0.05) whereas the affinity of Siglec-11/Fc fusion protein tended to be unchanged (*p* > 0.05) as shown in Figure 9 and Figure 10.

## 3. Discussion

In the present study, we developed the transwell co-culture system to investigate the involvement of Siglec checkpoint axis in the interplay between glioma and immune cells exposed to the conventional drugs for management of malignant gliomas. Standard treatment for glial tumours of high-grade malignancy contains cytoreductive surgery followed by adjuvant radio- and chemotherapy [28]. TMZ is a drug of choice responsible for cytotoxic action in malignant cells related to DNA hypermethylation, however, the intrinsic or acquired resistance of glioma cells is also observed [29]. In addition to TMZ, standard therapy provides administration of Dex as a part of preoperative and postoperative management aimed at limiting tumour-related oedema as well as adverse effects of radiochemotherapy [30,31]. Besides the therapeutic benefits, there is increasing evidence that Dex, particularly in high doses, may reduce overall- and progression-free survival (OS and PFS, respectively) of gioblastoma multifome (GBM) patients but the mechanisms underlying this clinical observation are still largely unknown [23,32]. The therapeutic pitfalls in this field have been also suggested when concomitant Dex and TMZ were used. The retrospective review by Shields et al. revealed that the use of Dex in patients receiving TMZ was a poor prognostic factor of OS and PFS [24]. The efficacy of TMZ-based therapy was determined in multiple in vitro studies. It has been shown that Dex can play critical function in prevention of TMZ-induced cytotoxicity. In U87MG and T98G glioma cultures, Dex antagonized the TMZ-induced upregulation of proapoptotic mediators, such as intracellular Ca^2+^, caspase-3, calpain and Bax, resulting in counteractive effect on cell death and viability [33,34,35]. It is of particular importance in the context of undesirable clinical outcome in the field of glioma therapy. Moreover, the recent correlative studies by Wong [36] and Grossman [37] suggest that Dex-induced immunosuppression decreases efficacy of standard therapies and strongly affect the patient’s antitumour immunity. It has been shown that Dex-induced severe reduction of CD4^+^ immune cells was associated with high tumour aggressiveness, more rapid disease progression and shorter survival [38]. Finally, several alkylating drugs exert the immunogenicity-increasing effect in glioma cells and thereby facilitate the immune cells activation, however, the concomitant dosage of Dex seems to block this benefit [39,40,41].

Since the sialoglycans-Siglecs interactions are involved in the suppression of effector immune cells activity, their role in cancer biology is extensively studied. Based on our previous investigations [26,27] that confirmed an impact of glucocorticosteroids on Siglecs expression, we aimed to determine the potential role of TMZ/Dex in the modulation of immune Siglec-based checkpoint in the in vitro model of glioma. Here, we hypothesized that both, Dex and TMZ, may affect immune surveillance by mechanisms linked to altered sialoglycans and their recognition by Siglecs. For experiments, we used glioma cells of different sensitivity, as defined in independent studies according to the half maximal inhibitory concentration (IC_50_) value, O^6^-methylguanine–DNA methyltransferase (MGMT) expression, wild type p53 expression and phosphatase and tensin homolog (PTEN) deficiency [42,43]. Despite the low MGMT expression suggests sensitivity to TMZ in both cell lines, the U87MG cells showed higher IC_50_ suggesting partial resistance when compared to A172 cells [44]. The immune response to pharmacological stimulation and interplay with glioma cells was observed in non-adherent THP-1 and adherent HMC3 cells. The assessment of immune activity by measurement of concentration of pro- and anti-inflammatory cytokines revealed interesting differences between tested cells. In response to pharmacological stimulation, the expected effects in both monoculture THP-1 and HMC3 cells were observed. As shown, the strong anti-inflammatory potential of Dex was expressed by a reduced level of proinflammatory cytokines, whereas anti-inflammatory IL-10 was enhanced. In opposite, the exposure to TMZ induced different effects on cytokines expression in both monocultures. Moreover, adherent HMC3 microglia cultured alone showed several fold higher immune activity than THP-1 monocytes, whereas the reverse effect was observed in co-culture systems. The participation of glioma and immune cells in cytokine production and release in transwell co-culture system has not been fully analysed and is the limitation of this study. However, given the higher cytokine production and secretory activity of immune cells that glioma cells, we can suggest that immune cells are the main donors of detected cytokines. In presence of glioma cells, the immune mediators-producing capability of THP-1 monocytes was markedly higher than HMC3 microglia. Similarly to monocultures, the inhibitory action of Dex was also observed in co-cultured immune cells. Interestingly, the opposite effects of TMZ on production of pro-inflammatory cytokines in THP-1 and HMC3 cells have been also observed. Our results suggest that infiltrating monocytes and resident microglia may respond differentially to external stimuli within the brain. Additionally, the missed assessment of cytokine profile in glioma cells is a weakness of this study. The analysis of cytokine production and release in A172 and U87MG cells, including factors that induce an M2-like microglial cell phenotype such as IL-10, IL-6, IL-4 and TGF-β may be an interesting observation in the future studies. According to Yamasaki et al., the different phenotypes and effector activity of immune cells can be reflected in different gene expression profiles due to distinct developmental origin and renewal mechanisms [45]. The detailed profiling of resident microglia and infiltrating monocytes revealed differences in mRNA, miRNA, and protein expression. The analysis by Butovsky et al. has identified characteristic microglia signature dependent on specific transcription factors and miRNA but not expressed in peripheral monocytes [46].

To determine the impact of Dex/TMZ treatment on immune function of THP-1 monocytes and HMC3 microglial cells, the M1/M2 polarization status was investigated in accordance with the intracellular expression of Arg-1 and Arg-2. Phenotypic plasticity of microglia and macrophages is one of the hallmarks featuring functional engagement of the immune system in the promotion and progression of pathology. The M1 phenotype is characterized by the secretion of high levels of pro-inflammatory factors associated with initiation and sustaining of inflammation. In the central nervous system (CNS), the M1 cells are the predominant population within the injury area that promotes neuroinflammation in neurodegenerative disorders [47]. The M2 cells, which have opposite function to that of M1, are characterized by their involvement in promotion of tumor growth, survival, and metastasis [48]. Since arginase isoenzymes (Arg) are known as regulators of M1/M2 phenotypes, the expression of Arg-1 is a hallmark of suppressive M2 macrophages [49]. The function of Arg-2, as mitochondrial enzyme, is closely linked to enhanced reactive oxygen species (ROS) generation resulting in cellular pro-inflammatory response [50,51]. In the present study, the exposure to Dex, was accompanied by enhanced Arg-1 expression in THP-1 cells. Additionally, this effect was strengthened in the presence of glioma cells. This observation is not surprising, since glucocorticosteroids, including Dex, are known to elicid M2 phenotype through direct upregulation of Arg-1 activity [52,53]. Interestingly, in response to TMZ, both THP-1 and HMC3 cells, showed enhanced Arg-2 expression suggesting M1 polarization as confirmed by high level of pro-inflammatory IL-8, IL-1β and TNF-α. Similar effect of TMZ on IL8 expression has been noted by Hasan et al. [54]. Furthermore, the antagonizing impact of Dex on TMZ action was observed in this study. In most cases, TMZ potentiated immune response in the co-culture systems, whereas the concomitant treatment with Dex caused the intense worsening in cellular cytokine production in both monocytic THP-1 and microglial HMC3 cells. This observation can be completed with an analysis of the phagocytic activity of the tested cells in future studies. The obtained results suggest that standard anti-glioma therapy may initiate the “danger” signals and exert anti-tumour effect by providing appropriate cytokine milieu. Nevertheless, our study suggests that the use of Dex according to the regimen of treatment may result in deterioration of immune surveillance in the brain.

The glioma microenvironment is composed of mixed populations of myeloid cells, including infiltrating macrophages and resident microglia that constitute approximately 80% and 20% of the total population, respectively [55,56]. These cells display predominantly suppressive phenotype attributed the elevated production and release of anti-inflammatory cytokines and growth factors, thereby promoting tumour growth and invasive potential. The targeting glioma cellular pathways, such as the phosphoinositide 3-kinase (PI3K)/protein kinase B (AKT), the p53 pathways, and epidermal growth factor receptor (EGFR) gene amplification or mutation, have failed to show their clinical efficacy due to the high activity of compensatory mechanisms, blood brain barrier selective vulnerability and poor tolerability and safety [57,58]. Despite the progress in the field of immunobiology it is not fully understood what regulatory systems are responsible for the interplay between glioma and immune cells. As described previously, the expression of several checkpoints in brain tumours, including programmed cell death protein 1 (PD-1) and cytotoxic T-lymphocyte-associated protein 4 (CTLA-4), is comparable to that observed in non-CNS origin malignancies.

However, the targeting of immune checkpoints as candidates of new therapeutic strategy in gliomas management remains controversial. Despite the promising efficacy preclinical data, no benefits in overall survival have been shown in the early clinical trials [59]. Given limited availability and therapeutic efficacy of immune-related drugs, TMZ is used in the gliomas first-line therapy since 2005 [21,60]. On the other hand, recent studies focus on new approaches to increase the efficacy of PD-1/PD-L1 and CTLA4 inhibitors in brain tumours [61]. In contrast, the newest observation by Iorgulescu et al. showed that the addition of Dex to anti-PD-1 therapy resulted in reduced survival [62]. Based on the evidence that standard drugs interfere with checkpoint-related pathways, we explored the effects of TMZ/Dex on Siglec-sialic acid axis.

Since aberrant sialylation was found in different types of malignancies, the interplay between Siglec-expressing immune cells and sialoglycans within the tumour microenvironment is considered as a mechanism that forms immune surveillance in tumours. Among the human CD-33-related Siglecs, the engagement of Siglec-7 and Siglec-9 was confirmed in growth and progression of breast, pancreas and colon cancers [63,64,65]. The tumour-derived glycans present unique sialylation patterns and deliver specific ligands for Siglec receptors. In line, the binding of α2.3- and α2.6-linked sialic acids to Siglec-9-expressing neutrophils, NK cells and T cells results in the ITIM-mediated signal transduction and leads to the reduction of effector function [66,67,68]. In the brain, sialic acids are the essential components of glycoconjugates implicated in crucial cell-cell interactions underlying neuronal growth and migration, and synapse formation [69]. One of them, polysialylated neuronal adhesion molecule (PSA-NCAM), has been described as a marker of brain cancer progression as well as a modulator of brain immunity through Siglec-11 recognition in microglial cells [70,71]. Given “cis” recognition of ligands, Siglec-11-mediated suppression has been proposed as a mechanism involved in the maintenance of immune homeostasis in the brain, however “trans” interaction with PSA-NCAM underlie the impaired surveillance in the brain pathology [72]. The molecular and clinical analysis of Siglecs profile by Santegoets et al. revealed high expression of Siglec-3, -5, -7 and Siglec-9 in both monocytic and polymorphonuclear myeloid derived suppressor cells isolated from blood of glioma patients [73]. Additionally, the RNA analysis in these cells confirmed mRNA expression for Siglec-10, -11, -14 and Siglec-16. Similar to samples isolated from blood, the glioma infiltrating cells showed high expression of Siglec-5 and Siglec-9. The results obtained in our study seem to be in line with this observation. The THP-1 cells used in presented experimental model were originally derived from the peripheral blood in presented similar Siglec profile in both gene and protein level. Interestingly, Li et al. demonstrated the synergistic relationship between several Siglecs and immune checkpoints, including PD-1 and CTLA4. As presented data have shown, the low expression of Siglecs and corresponding immune checkpoints was closely correlated with better prognosis in patients. Therefore, it may suggest, that the combined therapy with Siglecs and immune checkpoints inhibitors may benefit the overall survival in patients with glioma [74].

Based on the immune status of THP-1 and HMC3 cells measured by cytokines level and Arg-1 and Arg-2 expression, we asked here about the potential role of Siglecs in Dex/TMZ-modulated immunity. For this purpose, we examined the paired Siglecs and coupled signalling molecules expression at the genes level as well as the protein level and the affinity of inhibitory counterparts to glioma cell surfaces. In both HMC3 microglial cells and THP-1 monocytes, we found the transcripts for *SIGLEC5*, *SIGLEC11*, *SIGLEC14*, and *SIGLEC16*. The quantitative real-time PCR revealed stimulatory impact of Dex and TMZ on *SIGLECs* transcripts in analysed cell populations. According to the cellular distribution of inhibitory counterparts, the expression of Siglec-5 and Siglec-11 protein was confirmed in THP-1 and HMC3 cell lines, respectively, by flow cytometry with the monoclonal antibody. As shown, the exposure to Dex caused an increase in cell surface Siglec-11 expression in both HMC3 monoculture and co-culture. The opposite effect was observed in response to TMZ. In THP-1 mono- and co-cultures the cell membrane distribution of Siglec-5 was increased in response to Dex, whereas modulatory effect of TMZ in this population was not observed. The Dex-induced changes in Siglec-11 expression were accompanied by M2 polarization as described above. Interestingly, the elevated expression of Arg-1 protein in HMC3 cells was in line with high *PTPN6 (SHP1)* and *PTPN11 (SHP2)* expression found on the mRNA level. It may confirm the immunosuppressive status of these cells. In the field of Siglecs assessment, there are several limitations of this study. First, we have used specific monoclonal antibody which recognizes extracellular domain of inhibitory Siglec-5 but may also show cross-reactivity to activatory Siglec-14. Given the predominant Arg-1-related M2 phenotype and upregulated *PTPN6* and *PTPN11* transcripts levels, we conclude that the observed changes are rather inhibitory. Second, the Siglec-11 antibody confirmed the expression of Siglec-11 on the membrane of HMC3 cells, although the low level of *SIGLEC11* transcripts was detected. This phenomenon can be linked to multiple transcript variants encoding different isoforms found for this gene [72]. Wang and Neumann have shown that human microglia express short splice variant 2 that has different molecular properties [72,75]. Moreover, it has been suggested that the splice variants of *SIGLEC11* are also differentially transcribed in distinct tissue types [76]. Therefore, the commercial primer used in our study for *SIGLEC11* assessment in HMC3 cells showed a limited diagnostic capacity that may explain differences in *SIGLEC11* transcript levels in HMC3 and THP-1 cells [72]. The results of our study seem to be in conformity with the bioinformatic analysis by Chen et al. [76]. They showed that *SIGLEC* family genes are differentially expressed in tissues of various cancers. Among highly aggressive human tumors, glioblastoma multiforme expressed the most of *SIGLEC* genes. Interestingly, while Siglec-11 mRNA expression decreased in most tumours, the up-regulation in glioblastoma multiforme was observed [76]. This is of particular importance due to genes-dependent human phenotypes featured by variable expression of paired Siglecs. The loss of Siglec-14 linked to *SIGLEC-5/14* fusion polymorphism correlates to lower cytokines expression in macrophages compared to the levels found in cells from *SIGLEC-14 ^+^/^+^* individuals [77,78]. In line, the function of Siglec-14 in individuals with *SIGLEC-5/14^+/+^* genotype is to counteract the suppressive effects of Siglec-5-mediated signalling pathways [78]. This dependence was described in invasive and inflammatory diseases, but the data on the function of paired Siglecs in malignancies remains highly limited [79,80,81]. The previous studies suggest that glucocorticosteroids may participate in suppression of immune cell subsets by upregulating CD-33-related Siglecs expression [26,27,82]. Here, the impact of Dex and/or TMZ on the sialylation pattern has not been investigated. However, changes in the binding of recombinant Siglec-5/Fc and Siglec-11/Fc proteins to glioma cells reflect the sialic acid-related alteration in cell membranes. The proper sialylation of mammalian glycoconjugates results from the balanced expressions and activities of sialyltransferases and sialidases involved in attachment or cleavage of sialic acids from the sugar chains of glycolipids and glycoproteins [83]. In cancer, the aberrant sialylation is closely related to elevated expression of sialyltransferases, including ST3Gal1, STGal4, ST6-Gal1 and ST8Sia2, and their products, especially Sialyl-Levis^a,x^ epitopes that correlate with poor prognosis [84,85]. Indeed, the ST8Sia2-mediated aberrant polysialylation in selected cancers, including glial tumours, is known to negatively regulate the production of pro-inflammatory mediators [86]. In addition, the sialylation pattern undergoes dynamic changes in response to intrinsic regulatory mechanisms, however the effects of external stimulation have been also observed. At cellular level, the exposure to Dex exerts the opposite outcomes on sialoglycans-related enzymes, but an influence of TMZ was not studied [87,88]. In this study, the quantitative flow cytometric analysis detected differences in Siglec/Fc proteins binding in monocultured and co-cultured glioma cells. Additionally, the exposure to Dex and/or TMZ increased the affinity of Siglec-5/Fc and Siglec-11/Fc proteins in both, A172 and U87MG cells. Given the experiments were performed in transwell system, the observed enhancement in immune status and sialome-Siglec interaction were limited to physically independent cellular interactions but closely related to the exposure to Dex and/or TMZ as well as soluble cell-derived mediators potentially involved in the regulation of diversity of immune cell phenotypes. The engagement of mixed glioma-microglia monolayer cultures should be the next observation of the involvement of Siglec checkpoint in cellular direct interactions. Moreover, previous studies have shown that the structure of membrane glycoconjugates is highly variable and depends on the phase of cell division [89,90]. The estimation and comparison of sialylation and related changes in Siglecs recognition may be interesting in the range of different cell cycle phases of tested glioma cells. Several studies showed that selectin *p* ligand (PSGL-1, CD162) and PSA-NCAM are potential ligands for Siglec-5 and Siglec-11, respectively [91]. Therefore, further studies need to evaluate the interaction between glioma sialylated ligands with inhibitory Siglec-5 and -11 on microglia and/or monocytes in the tumour microenvironment and resulting functional consequences. According to Chen et al., the *SIGLEC* family genes are implicated in the infiltration of immune cells, including macrophages, in the tumour microenvironment as well as correlate to markers of immune activity, including chemokines axis [76]. These functions seem to be closely related to invasive tumours biology and thereby potentially associated with prognosis and the patient’s overall survival. According to the recent advances in immunology, immune checkpoints play pivotal role as regulators of cancer progression and potential targets for immunotherapy. In the field of glioma biology, the risk to benefit ratio of conventional therapies is under investigation in patients with brain malignancies. The present study suggests that Dex and TMZ-based therapies, as standard management in gliomas, can exert a modulatory effect on the host immunity within the tumour microenvironment. The observed effects in immune functions in microglia and monocytes were diverse and, importantly, dependent on cell type and origin. Our data seem to confirm findings by Kaminska et al. that microglial cells attracted by glioma display high plasticity and adapt tumour-created conditions in microenvironment by changes in cellular phenotype ranging from pro-inflammatory to alternatively activated [92]. The paired Siglecs, depending on the patient’s genotype, may result in inhibitory or activatory signal transduction underlying the mechanisms of immune surveillance. Given the opposite effects of Dex and TMZ on immune function, including modulation of Siglecs expression in the gene and protein levels, the characteristics of patients by the engagement of several Siglec receptors and related molecular mechanisms may be useful in verifying therapy and prediction of overall survival.

## 4. Material and Methods

### 4.1. Cell Cultures, Co-Cultures and Treatment

Both, U87MG and A172 human glioma cells and HMC3 human microglia were obtained from ATCC and maintained in Eagle’s minimal essential medium (EMEM, ATCC) with 10% heat-inactivated foetal bovine serum and 100 μg/mL penicillin/streptomycin (all Gibco; Thermo Fisher Scientific, Inc., Waltham, MA, USA). The non-adherent human monocytic cell line THP-1 (ATCC, TIB-202) was cultured in RPMI-1640 (ATCC) supplemented with 10% heat-inactivated foetal bovine serum, 1% penicillin/streptomycin mixture and 2-mercaptoethanol (Gibco, Thermo Fisher Scientific, Inc., Waltham, MA, USA) to a final concentration of 0.05 mM. All cell types were kept in culture at 37 °C in a humified atmosphere containing 5% CO_2_ until passage 10 and analysed from passages 7–10. For experiments, the monocultured cells were plated in 6 well plates at a seeding density 0.3 × 10^6^/1.5 cm^2^ and cultured to reach confluency of 80%. The co-culture experiments were performed using the transwell culture system. Naïve THP-1 and HMC3 cells were cultured in six well plates, and naïve glioma cells were added to the inserts placed in the upper part of each well at a ratio 1:3 (microglia/macrophages:glioma). The lower and upper transwell sections were separated by a 0.4 µM microporous membrane to prevent close contact between glioma and immune cells. Both monocultured and co-cultured cells were exposed to TMZ (Merck, Darmstadt, Germany) and/or Dex (Dexaven, PharmaSwiss, Prague, Czech Republic) for 24 h. Before the treatment, TMZ was freshly dissolved in sterile dimethyl sulfoxide (DMSO, Merck) at a concentration of 0.053 M. The stock solution was added to the culture medium to obtain final concentrations of 10 and 50 μM known to cause the hypermethylating effect whereas the Dex concentration of 10 μM was defined as high clinical level [93,94,95,96].

### 4.2. Immune Status and Siglecs Expression in THP-1 and HMC3 Cells

For the M1/M2 phenotype determination, naïve and Dex/TMZ-treated immune cells were harvested for flow cytometry. Arginases are the arginase metabolizing enzymes closely linked with macrophages metabolism phenotype. Whereas arginase 2 (Arg2) represents M1 phenotype, the Arginase 1 (Arg1) can be considered as M2 marker [97,98]. Cells were diluted to 10^5^ per sample and incubated with Arg1 or Arg2 antibodies (both Invitrogen, Carlsbad, CA, USA; 2.6 μg/mL) for 30 min at 4 °C. To facilitate intracellular staining, cells were permeabilized in 0.1% Triton X-100 in 1x PBS for 10 min. at room temperature. Cells were washed with phosphate buffered saline, stained with appropriate secondary fluorescent antibody and analysed on Becton Dickinson flow cytometry system. As a negative control, the corresponding isotype control antibody was used. The median fluorescence intensity was calculated using Flowing Software (Turku Center for Biotechnology, Turku, Finland). For cytokine level assessment, the Cytokine Bead Array (CBA) Human Cytokine Kit (Beckton Dickinson Biosciences, San Jose, CA, USA) has been used. The IL-8, IL-6, IL-1β, IL-12p70, IL-10 and TNFα protein levels were quantitatively measured in the collected immune cells samples according to the manufacturer’s protocol. Briefly, 50 µL of assay beads, 50 µL of sample, or standard and 50 µL of PE-labelled antibodies (Detection Reagent) were added consecutively to each sample tube. The samples were incubated at room temperature for 3 h, washed with Wash Buffer, centrifuged and the resulting pellet was re-suspended in Wash Buffer. The resulting samples were analysed using a BD FACSCanto II flow cytometer and FCAP Array v3 software (both BD Biosciences Systems, San Jose, CA, USA). The inhibitory counterparts of human paired Siglec-5/14 and Siglec-11/16 have been investigated according to their distribution in monocytic THP-1 and microglial HMC3 cells, respectively [90]. Cells were analysed by flow cytometry after incubation with primary Siglec-5/14 and Siglec-11 antibody (both R&D Systems, Minneapolis, MN, USA; 5 µg/mL)

### 4.3. Real-Time PCR

Total RNA from collected THP-1 and HMC3 cells was purified using RNeasy Mini Kit (Qiagen; Hilden, Germany) following the classical described protocol. The ratio of absorbance at 260 nm and 280 nm was used to assess the purity of isolated RNA. In all samples 1 μg of total RNA was used to conduct the reverse transcription reaction by the SuperScript First-Strand Synthesis System for RT-PCR (Invitrogen) according to manufacturer’s instruction in the MJ Research Thermal Cycler (PTC-200, Watertown, MA, USA). Levels of primary transcripts of human genes: *SIGLEC5*, *SIGLEC14*, *SIGLEC11*, *SIGLEC16*, *TYROBP* (formerly *DAP12*), *PTPN6* (formerly *SHP1*) and *PTPN11* (formerly *SHP2*) were assessed by real-time PCR using commercial primers Qiagen QuantiTech Assay: HS_SIGLEC5_1_SG; HS_SIGLEC14_1_SG; HS_SIGLEC11_1_SG; HS_SIGLEC16_1_SG; HS_TYROBP_1_SG; HS_PTPN6_1_SG; HS_PTPN11_1_SG and HS_GAPDH_1_SG as a normalizer. The analysis was performed using the QuantiTech SYBR Green PCR Master Mix (Qiagen) following the manufacturer’s instruction and carried out in the CFX96 Real-Time PCR Detector (Bio-Rad, Hercules, CA, USA). Melting curve analysis was performed at the end of all reactions for exclusion of all nonspecific PCR products. To calculate of our data, we used the comparative C_T_ method for relative quantification for all gene transcripts.

### 4.4. The Binding Capacity of Siglec Receptors to Glioma Cells

To investigate the potential changes in cell surface sialylation and their importance in the cellular interplay through the inhibitory counterparts of paired Siglec-5/14 and Siglec-11/16 receptors, the binding ability of soluble Siglec proteins to the glioma cell surface has been assessed. Both, U87MG and A172 glioma cells were incubated with recombinant human Siglec-5/Fc and Siglec-11/

### 4.5. Statistical Analysis

The experimental data were compared using one-way ANOVA with a Bonferroni post-test using Instat (GraphPad Software Inc., San Diego, CA, USA). For each group, at least three independent experiments were performed. Results are expressed as a median fluorescence intensity (MFI) ± SD. Statistical differences were deemed at *p* < 0.05.

## 5. Conclusions

One of the major findings of this study that both Dex and TMZ alter glioma microenvironment immunity focuses on the immunomodulatory role of conventional drugs and its clinical importance in glioma therapy. The corresponding changes in cytokine production and release, and immune cell phenotypes were different in Dex and TMZ treated cells. The observed opposite effects of Dex towards TMZ in concomitant therapy should raise a caution during the high dose steroid management. Additionally, the finding that expression of inhibitory counterparts of paired Siglecs in immune cells and their altered affinity to the glioma cell surface in response to pharmacological stimuli may suggest clinically useful value in the verification and prediction of therapy efficiency in patients with different immune response phenotypes.

## Data Availability

The data presented in this study are available on request from the corresponding author.
